# Immature leukocyte and plasma-induced cell death reveal subclinical immune activation in EGPA patients in remission

**DOI:** 10.1007/s00011-026-02189-7

**Published:** 2026-02-02

**Authors:** Chiara Baggio, Luca Iorio, Carlotta Boscaro, Federica Davanzo, Veronica Davanzo, Michela Pelloso, Marta Tonello, Paolo Sfriso, Mattia Albiero, Roberta Ramonda, Andrea Doria, Roberto Padoan, Francesca Oliviero

**Affiliations:** 1 Rheumatology Unit, Department of Medicine - DIMED, University of Padova, Padua, Italy; 2 Department of Medicine - DIMED, University of Padova, Padua, Italy; 3 Laboratory of Experimental Diabetology, Veneto Institute of Molecular Medicine, Padua, Italy; 4 Department of Laboratory Medicine, University-Hospital of Padova, Padua, Italy; 5 Department of Surgery, Oncology, and Gastroenterology, University of Padova, Padua, Italy

**Keywords:** ANCA-associated vasculitis, Eosinophilic granulomatosis with polyangiitis, Inflammasome, Cytology, Innate immune cells, Neutrophils

## Abstract

**Objective:**

ANCA-associated vasculitis (AAV) is a group of rare autoimmune diseases characterized by pauci-immune necrotizing inflammation of small to medium-sized blood vessels, in which ANCAs targeting neutrophil antigens promote neutrophil activation, endothelial injury and organ damage. Although AAV follows a relapsing-remitting course, the immune landscape during remission remains poorly defined. This study investigated leukocyte alterations across AAV subtypes and examined whether plasma from ANCA-positive and ANCA-negative eosinophilic granulomatosis with polyangiitis (EGPA) patients in remission modulates peripheral blood mononuclear cell (PBMC) responses in healthy donors (HDs).

**Methods:**

Peripheral blood was collected from 62 AAV patients in remission and 28 age- and sex-matched HDs. Leukocyte morphology was assessed via May-Grünwald Giemsa staining. Circulating cytokines and chemokines were quantified by ELISA. HD-derived PBMCs were exposed to plasma from EGPA patients, antiphospholipid-positive controls, or HDs. Cell death, metabolic activity, and cytokine production were evaluated using Trypan Blue, Annexin V/PI staining, MTT assays, and ELISA. Chemotaxis assays assessed cell migration in response to conditioned media, with or without Anakinra or CCR1 inhibitor J113863.

**Results:**

AAV patients showed increased immature neutrophils. Plasma from ANCA-positive EGPA patients induced PBMC death, inflammasome-related cytokine release, and secretion of chemotactic and proangiogenic factors. Conditioned media enhanced immune cell migration in a cytokine-dependent manner.

**Conclusion:**

These findings indicate persistent subclinical immune activation during AAV remission, particularly in ANCA-positive EGPA, suggesting a role for mononuclear cell-mediated inflammation in relapse risk and the potential utility of immune monitoring.

**Supplementary Information:**

The online version contains supplementary material available at 10.1007/s00011-026-02189-7.

## Introduction

Anti-neutrophil cytoplasm antibody (ANCA)-associated vasculitis (AAV) encompasses a group of rare chronic autoimmune disorders characterized by necrotizing inflammation of small- to medium-sized blood vessels and multisystem organ involvement. AAV patients are classified into granulomatosis with polyangiitis (GPA), microscopic polyangiitis (MPA), and eosinophilic granulomatosis with polyangiitis (EGPA) [1, 2]. ANCAs, which primarily target neutrophil and monocyte cytoplasmic enzymes such as myeloperoxidase (MPO) and proteinase-3 (PR3), are detected in approximately 90% of patients with GPA or MPA, and in 30–40% of EGPA patients [1, 3].

EGPA exhibits a distinct immunopathological profile compared to GPA and MPA, characterized by overlapping features of vasculitis and eosinophilic inflammation [4]. Genetic studies have revealed divergent genetic architectures for ANCA-positive and ANCA-negative EGPA patients, suggesting fundamentally different underlying pathobiological mechanisms [5]. ANCA-positive EGPA shares genetic susceptibility and clinical features more closely aligned with GPA/MPA, while ANCA-negative EGPA predominantly manifests with eosinophil-driven, asthma-related features [6].

During active phases of AAV, neutrophils are the primary effector cells, mediating vascular damage through ANCA-driven activation, degranulation, reactive oxygen species generation, and formation of neutrophil extracellular traps (NETs), contributing to endothelial injury and vascular inflammation [1, 7]. Although less defined, monocytes and lymphocytes are increasingly recognized as active contributors to vascular inflammation, complementing neutrophil-mediated damage by releasing pro-inflammatory cytokines, presenting antigens, and sustaining immune cell crosstalk [8–13]. In addition, accumulating evidence indicates that, in AAV, immunogenic cell death generates DAMP that not only sustain neutrophil activation but also critically engage the mononuclear compartment. Monocytes respond to these danger signals through inflammasome activation, enhanced IL-1β release, and defects in immune-checkpoint regulation, while T and B lymphocytes are activated by increased antigen availability and persistent danger signaling. Together, these processes promote loss of immune tolerance and support the development of pathogenic ANCA responses [14].

Although a minority of patients achieve long-term remission off therapy, remission in most AAV patients appears “quiet” rather than “off,” with residual immune activation potentially predisposing individuals to relapse or cumulative organ damage [15]. Understanding the immunological status during remission is therefore a crucial unmet need. In particular, research on ANCA positivity and its implications for subclinical inflammation or disease progression in EGPA remains limited.

Importantly, studies suggest that even during clinical remission, persistent immune activation is detectable. Recent studies have highlighted ongoing biochemical and functional evidence of neutrophil priming and NETosis even in clinically quiescent disease, suggesting a state of persistent innate immune activation during remission [16, 17]. Additionally, peripheral blood mononuclear cell (PBMC)-focused investigations indicate sustained adaptive and innate immune dysregulation during remission, characterized by persistent monocyte activation, impaired immune checkpoint modulation, and autoreactive B-cell potential, further supporting the notion of subclinical immune activity [18–20].

However, comprehensive studies assessing leukocyte profiles and their alterations in patients during remission are still lacking. To address these knowledge gaps, in line with previous efforts to identify leukocyte signatures associated with treatment response in AAV [21], our study aims to define leukocyte morphological and maturation features, nuclear abnormalities, and cell death, to uncover subclinical inflammation in patients in clinical remission. Furthermore, based on preliminary findings of residual immune activation and cell death in ANCA-positive EGPA patients, in vitro assays using PBMCs from healthy donors were designed to investigate the effects of plasma from these patients. We explored whether plasma from ANCA-positive and ANCA-negative EGPA patients in remission could modulate healthy donors PBMCs inflammatory responses and associated cell death. This investigation aimed at potentially identifying novel biomarkers and therapeutic targets for improved disease monitoring and management strategies.

## Materials and methods

### Patient cohort and blood sample collection

Blood samples were collected from 62 AAV patients (17 GPA, 31 EGPA, 14 MPA) attending the Padova Vasculitis Center of Padova University Hospital and classified according to the EULAR/ACR criteria [22]. All patients were in stable clinical remission for at least one year [22]. Clinical remission was defined as absence of new or worsening vasculitis manifestations and BVASv3 = 0 at serial assessments, with C-reactive protein and ESR within or close to the normal range. Demographic data and clinical features of the AAV cohort are reported in Table 1. All patients had been off oral or intravenous glucocorticoids for ≥ 4 weeks [23] and, at the time of sample collection, were not treated with any conventional DMARDs (cDMARDs), such as methotrexate, cyclophosphamide and mycophenolate mofetil, to exclude potential influence on leukocyte count or morphology [24–26]. Patients received, as maintenance therapy, only rituximab or anti–IL-5/IL-5 receptor agents, which were the sole treatments permitted at the time of sample collection. Patients with ongoing infection, malignancy, other autoimmune diseases, organ transplantation, dialysis or plasmapheresis were also excluded. Twenty-eight samples from healthy donors (HDs) were also collected, they were chosen among healthcare workers of Padova University Hospital and were not affected by relevant comorbidities. Patients and controls did not differ significantly in age (Mann–Whitney test) or sex distribution (χ² test). All participants gave their written informed consent to the study. The collection of blood samples was conducted within the context of standard clinical care. The study was approved by the Ethics Committee of Padua University Hospital (protocol code: 6315/AO/25).

**Table 1:** Demographic data and clinical features of AAV patients

	GPA (*n* = 17)	EGPA (*n* = 31)	MPA (*n* = 14)	All (*n* = 62)	HD (*n* = 28)
Sex, *n* (%)					
Female	9 (53)	12 (39)	6 (43)	27 (43)	16 (57)
Male	8 (47)	19 (61)	8 (57)	35 (57)	12 (43)
Age at sample, years, m (IQR)	56 (45–69)	61 (56–71)	64 (52–73)	61 (53–72)	60 (41–62)
ANCA-positive at diagnosis, n %					None
ANCA	15 (88.2)	13 (42)	14 (100)	42 (68)	
PR3	12 (82.4)	0 (0)	3 (78.6)	15 (24)	
MPO	1 (1.7)	13 (42)	11 (21.4)	25 (40)	
Double positive	2 (11.8)	0 (0)	0 (0)	2 (3)	
ANCA-positive at sample, n %					None
ANCA	5 (8.3)	5 (16)	6 (42.9)	16 (26)	
PR3	4 (6.7)	0 (0)	1 (7.1)	5 (8)	
MPO	1 (1.7)	5 (16)	5 (35.7)	11 (18)	
Disease duration at sample, m (IQR)	52 (29–143)	78 (36–131)	95 (67–129)	72 (38–140)	None

Data are expressed as the median (m) and interquartile range (IQR). Abbreviations are as follows: AAV, ANCA associated vasculitis; ANCA, Anti-neutrophil cytoplasmic antibodies; GPA, granulomatosis with polyangiitis; EGPA, eosinophilic granulomatosis with polyangiitis; MPA, microscopic polyangiitis; MPO, myeloperoxidase; PR3, proteinase 3; IQR, interquartile range; n, number

### Reagents

May-Grünwald and Giemsa staining, PBS, DMSO, MTT, Trypan blue and Histopaque 1077 (Ficoll) were from Sigma-Aldrich (St. Louis, Missouri). ELISA kits for TNFα (sensitivity: 2 pg/mL), IL-1α (sensitivity: 0.6 pg/mL) and VEGF (sensitivity: 4.1 pg/mL) were from BioLegend (San Diego, California), total IL-18 (IL-18 and IL-18 bound to IL-18 binding protein, BP) (sensitivity: 11.7 pg/mL) and CCL-23 (sensitivity: 15.6 pg/mL) were from R&D Systems (Minneapolis), IL-1β (sensitivity: 0.3 pg/mL), and CyQUANT™ LDH Cytotoxicity Assay were from Thermo Fisher Scientific (Waltham, Massachusetts). LPS was from InvivoGen (Toulouse, France). FITC Annexin V Apoptosis Detection Kit I was from BD PharmingenTM (Franklin Lakes, New Jersey).

### Analyses in the whole AAV cohort (GPA, EGPA, MPA)

#### Leukocyte morphology and cytological evaluation

Blood samples from AAV patients (*n* = 62) and HDs (*n* = 28) were collected in EDTA tubes. May-Grünwald Giemsa (MGG) staining was used for studying cellular morphology and to perform a cytogenic evaluation of leucocytes [27]. Oil immersion microscopy using 1000× magnification was applied for the cytological analysis. Leukocytes were identified based on morphology. For each smear, at least 200 leucocytes were examined and the number of neutrophils, monocytes, lymphocytes, eosinophils and basophils were recorded as percentage. All smears were independently evaluated by two operators, and the final values reported represent the average of the two independent measurements. All slides were examined for the presence of vacuoles and different types of nuclear abnormalities including micronuclei (MNi), buds, binucleated cells, hypersegmented neutrophils, karyolitic, karyorrhectic, and pyknotic cells (Online Resource 1) [27–30]. Cell death morphology was identified according to classical criteria of cell shrinking, nuclear condensation, and fragmentation. Neutrophils were distinguished from lymphocytes based on acidophilic or basophilic cytoplasmic staining. Karyorrhectic cells were characterized by fragmentation and disintegration of the nucleus. Karyolitic cells, that represent a very late stage in the cell death process, stained uniformly with eosin due to the complete dissolution of the chromatin and appeared as a ghost-like image. A left shift indicates the presence of immature neutrophils (band neutrophils) in the blood. The Immature neutrophils/total neutrophils ratio (I/T) calculated by dividing the percentage of immature neutrophils by the percentage of total neutrophils (both immature and mature), allowed to classify patient group into normal (< 0.2), moderate shift to the left (0.2–0.29) and severe shift to the left (≥ 0.3) [31]. Conversely, the presence of hypersegmented neutrophils, with five or more nuclear lobes, described a right shift in cell population. A right shift is often seen as a normal response to recovery from an infection or inflammation [32].

#### Plasma collection and cytokine measurements

Blood sample, collected in EDTA tubes, were centrifuged for 5 min at 400 g and plasma was stored at −80 °C until further analysis. The following cytokines, chemokines and growth factor were measured in plasma from AAV patients using commercially available enzyme-linked immunosorbent assay (ELISA) kits: interleukin (IL)-1β, IL-18, IL-1α, TNFα, CCL-23 and VEGF.

### In vitro assays in the EGPA subgroup

#### PBMCs isolation and plasma stimulation assays

Blood was drawn in heparin-coated tubes from HDs (buffy coat, *n* = 5). PBMCs were isolated by density-gradient centrifugation using Histopaque 1077 (Sigma-Aldrich). PBMCs were seeded into 96-well plates at 2.5 × 10^5^ cells/well, in RPMI 1640 (Sigma-Aldrich) supplemented with 1% glutamine (Sigma-Aldrich) and 1% penicillin-streptomycin (Sigma-Aldrich). Cells were first primed with LPS (10 ng/mL; InvivoGen) for 2 h, then stimulated for 24 h with 10% plasma collected from each of the following groups (*n* = 5 per group): EGPA patients persistently positive for ANCA from diagnosis through the time of sample collection (ANCA+), EGPA patients initially ANCA-positive at diagnosis but negative at sample collection (ANCA+/−), EGPA patients persistently ANCA-negative (ANCA-), patients positive for IgG antiphospholipid antibodies (aPL) serving as disease controls, and HDs. The next day, MTT and Trypan Blue assays were performed. Supernatants and cell lysates were collected for the measurement of specific cytokines, chemokines, and growth factors by ELISA (IL-1β, IL-18, IL-1α, CCL-23, and VEGF), as well as for the LDH cytotoxicity assay. Supernatants were analyzed to quantify extracellular inflammatory mediators release, whereas cell lysates were used to assess intracellular cytokine content.

#### Cell metabolic activity (MTT assay)

PBMCs were treated with plasma from EGPA patients (*n* = 15), patients positive for aPL (*n* = 5) and HD (*n* = 5) as described in section “PBMCs isolation and plasma stimulation assays”. Four hours before the end of incubation, a 10 µl stock solution of 3-[4,5 dimethylthiazol-2-yl]-2,5 diphenyltetrazolium bromide (MTT, 5 mg/ml in PBS) was added to each well. Then, formazan crystals were dissolved in 100 µL dimethylsulfoxide (DMSO). MTT reduction was quantified by measuring light absorbance with a multilabel plate reader (Multiskan FC, Thermo Fisher Scientific) at 570–630 nm. MTT reduction was expressed as the raw optical density (OD) value and represents the mean value of three independent assays, performed in quadruplicate.

#### Cell membrane integrity (LDH cytotoxicity assay)

PBMCs were treated with plasma from EGPA patients (*n* = 15), patients positive for aPL (*n* = 5) and HD (*n* = 5) as described in Sect. 2.4.1. Lactate dehydrogenase (LDH) is a cytosolic enzyme that is released into the cell culture medium when the plasma membrane is damaged. The colorimetric assay CyQUANT LDH Cytotoxicity Assay provides a simple and reliable method for determining cellular cytotoxicity. 50 µL of each sample medium and 50 µL of 1X LDH Positive Control were transferred in a 96-well flat bottom plate in triplicate wells. Then 50 µL of Reaction was added Mixture to each sample well and the plate was incubated at room temperature for 30 min protected from light. Finally, 50 µL of Stop Solution was added to each sample well. LDH activity was quantified by measuring light absorbance with a multilabel plate reader (Multiskan FC, Thermo Fisher Scientific) at 490–680 nm. LDH activity was expressed as the raw optical density (OD) value and represents the mean value of three independent assays, performed in triplicate.

#### Cell viability assessment (Trypan blue assay)

PBMCs were treated with plasma from EGPA patients (*n* = 15), patients positive for aPL (*n* = 5) and HD (*n* = 5) as described in 2.4.1 section. After 24 h Trypan blue was added to the cell suspension to a final concentration of 0.2%. Cells excluding Trypan blue (viable cells) as wells as dead cells were counted under the microscope with a Bürker haemocytometer. The percentage of cell death was reported.

#### Apoptosis and necrosis assessment by flow cytometry

PBMCs were seeded into 6-well plates at 2 × 10^6^ cells/well. PBMCs were treated with plasma from EGPA patients (*n* = 15), patients positive for aPL (*n* = 5) and HD (*n* = 5) as described in 2.4.1 section. Flow cytometry analysis was performed using commercially available FITC Annexin V Apoptosis Detection kit (BD PharmingenTM). Staining with FITC Annexin V is used in conjunction with propidium iodide (PI). Cells were incubated with antibodies for 15 min at room temperature. After staining, live cells show no fluorescence (Annexin- / PI-), late apoptosis (Annexin V+ / PI+) cells show both green and red fluorescence and necrotic (PI+) cells show red fluorescence. Data were acquired with a FACSCanto (BD Biosciences) cytometer followed by analysis using FlowJo software (BD Biosciences).

#### Chemotaxis assay

Chemotaxis experiments were performed in a 48-well modified Boyden chamber (Neuro Probe, Gaithersburg, MD) using 5 μm nucleo-pore polyvinyl pyrrolidine-free polycarbonate filters. As chemotactic stimuli, conditioned medium from PBMCs culture were added to lower chambers (30 µL) after appropriate dilution (1:2 in RPMI 0% FBS supplemented with 1% penicillin-streptomycin). Upper chambers were filled with 50 µL PBMCs suspension from HDs (*n* = 5) (50,000 cells/50 µL in RPMI with 1% FBS and 1% penicillin) in the presence or absence of J113863, a CCR1 inhibitor (10 µM) or anakinra (0.1 µg/mL). After 1.5 h incubation at 37 °C, non-migrating cells on the upper filter surface were removed by scraping. The cells that had migrated to the lower side of the filter were stained with Diff Quik staining, and densitometry was performed using Image J. Each condition was performed in sextuplicate. Results are reported as optical density (OD) arbitrary units.

### Statistical analysis

The Shapiro-Wilk test was used to analyse the distribution of continuous variables, and variables with a non-normal distribution were presented as medians with the corresponding interquartile range (IQR). Kruskal Wallis followed by Dunnet post hoc tests were used for multiple comparisons. The differences between the two groups of patients were tested using the Mann-Whitney U test. The One-way Anova test was used to analyse the variables with a normal distribution. Spearman correlation analysis was used to determine the correlations. Statistical analysis was performed with GraphPad Prism 8 (GraphPad Software Inc., La Jolla, CA, USA). A p-value < 0.05 was considered significant.

## Results

### Leukocyte morphology in AAV during remission

**Fig. 1:**
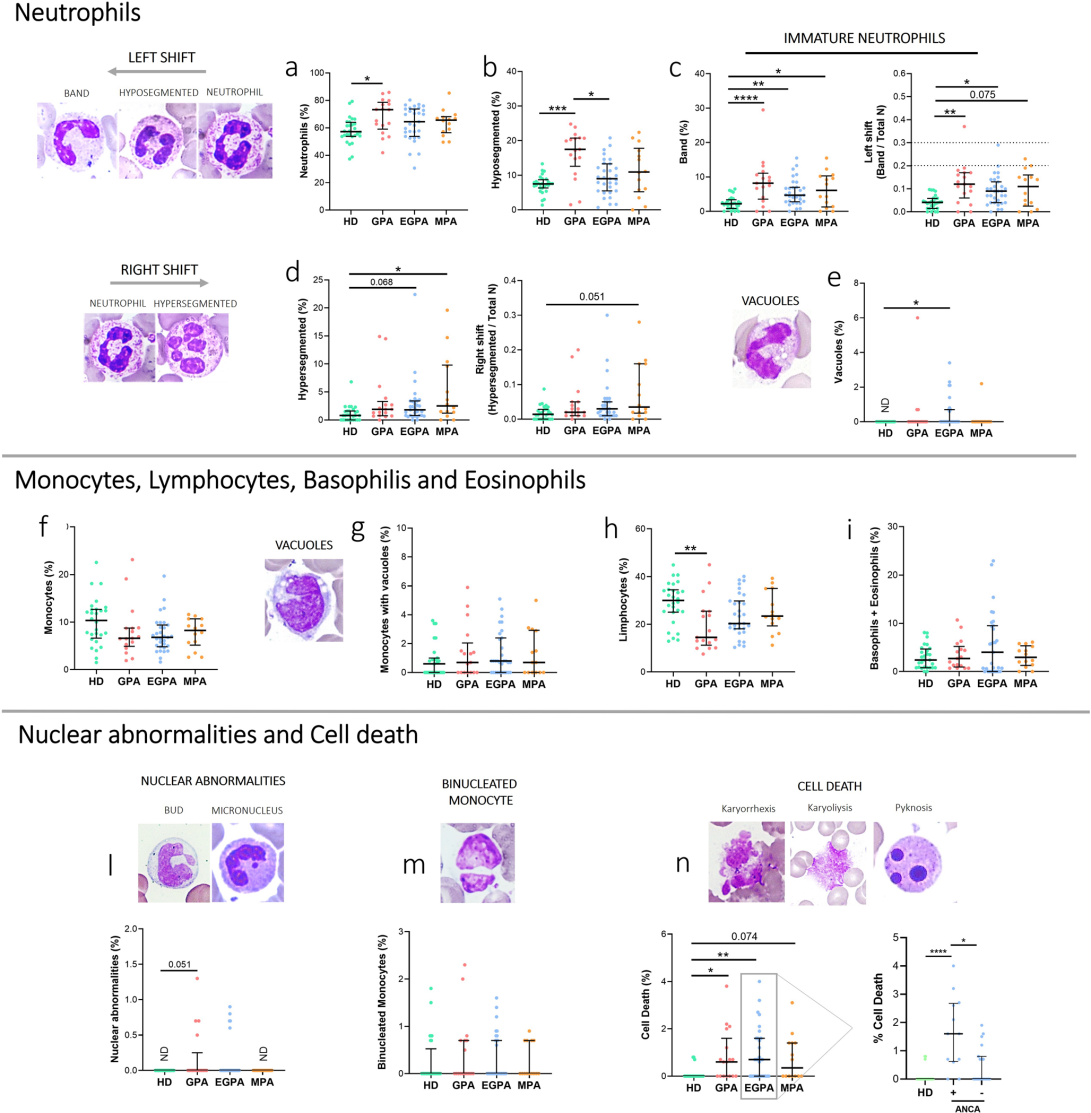
Cytological analysis in AAV patients. Peripheral blood smears from GPA (*n* = 17), EGPA (*n* = 31), MPA patients (*n* = 14) and HDs (*n* = 28) were stained using MGG staining. **a** Percentage of neutrophils **b** Percentage of hyposegmented neutrophils. **c** Percentage of band neutrophils and left shift index (I/T ratio). **d** Percentage of hypersegmented neutrophils and right shift index. **e** Percentage of neutrophils with vacuoles. **f** Percentage of monocytes. **g** Percentage of monocytes with vacuoles. **h** Percentage of lymphocytes. **i** Percentage of Basophils and Eosinophils. **l** Percentage of leukocytes with nuclear abnormalities. **m** Percentage of binucleated monocytes. **n** Rate of cell death. Data are shown as the median (IQR). p calculated according to the Kruskal-Wallis test. Dunn’s post hoc test: *p < 0.05, **p < 0.01, ***p < 0.001, ****p < 0.0001. Abbreviations are as follows: GPA, granulomatosis with polyangiitis; EGPA, eosinophilic granulomatosis with polyangiitis; MPA, microscopic polyangiitis; HD, healthy donors; IQR, interquartile range

We evaluated the percentage of neutrophils, their maturation stage, and cytological characteristics in all blood smears from patients with AAV. The percentage of neutrophils was significantly increased in GPA patients compared to HDs (*p* < 0.05) (Fig. 1a). GPA patients also showed higher percentages of hyposegmented neutrophils (pseudo-Pelger-Huët-like morphology) compared to EGPA (*p* < 0.01) and HDs (*p* < 0.001) (Fig. 1b). The percentage of band neutrophils was higher in GPA (*p* < 0.0001), EGPA (*p* < 0.01) and MPA (*p* < 0.05) compared to HD (Fig. 1c). A moderate left shift (I/T ratio) was observed only in 4 AAV patients (6.5%) (I/T ratio: 0.2–0.29), although the I/T ratio was higher in GPA (*p* < 0.01), EGPA (*p* < 0.05) and MPA patients (*p* = 0.075) compared to HDs (Fig. 1c). Conversely, hypersegmented neutrophils (right shift) were more frequent in EGPA (*p* = 0.068) and MPA (*p* < 0.05) than in HDs (Fig. 1d). Neutrophils vacuolization was present in 16% of AAV patients, with EGPA patients showing significantly more vacuolated neutrophils than HDs (*p* < 0.05) (Fig. 1e).

No significant differences were observed in the monocyte percentages and monocyte vacuolization rates across patient groups (Fig. 1f, g). The percentage of lymphocytes was significantly lower only in GPA patients compared to HDs (*p* < 0.05) (Fig. 1h). Eosinophil and basophil counts did not differ significantly across groups (Fig. 1I).

Regarding nuclear abnormalities (buds and micronuclei) were observed in 24% of GPA and 14% of EGPA patients but were absent in HDs (Fig. 1l). Binucleated monocytes were equally distributed among groups (Fig. 1m). The rate of cell death (pyknosis, karyolysis and karyorrhexis) was increased significantly in GPA (*p* < 0.05) and EGPA (*p* < 0.01) compared to HDs, with a trend toward higher rates in MPA (*p* = 0.074) (Fig. 1n).

Within the EGPA subgroup, ANCA-positive patients showed a significant increase in the percentage of cell death compared to ANCA-negative patients (*p* = 0.007) (Fig. 1n), and monocyte vacuolization was observed in ANCA-positive patients when compared to ANCA-negative patients (*p* = 0.03) (Online Resource 2).

### Circulating cytokines, chemokines and VEGF in AAV

**Fig. 2:**
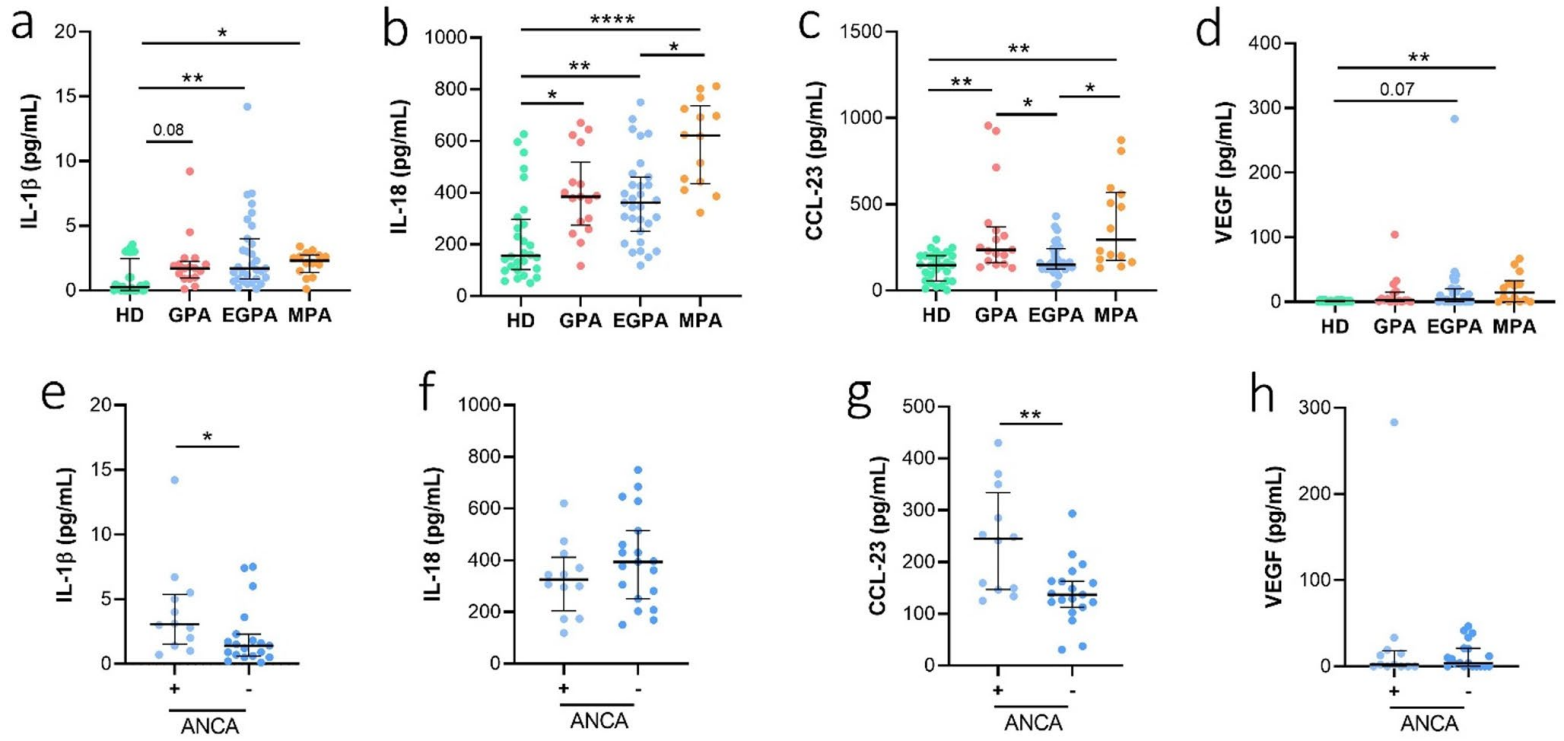
Circulating cytokines, chemokines and VEGF in AAV. Plasma samples from GPA (*n* = 17), EGPA (*n* = 31), MPA patients (*n* = 14), and healthy donors (*n* = 28) were analyzed for cytokine, chemokine, and VEGF levels using ELISA. **a** IL-1β levels. **b** IL-18 levels. **c** CCL-23 levels. **d** VEGF levels. Data are expressed as the median and interquartile range (IQR). p was calculated according to Kruskal Wallis test followed by Dunn’s post hoc test: *p < 0.05, **p < 0.01, ****p < 0.0001. Analyses in EGPA patients were further stratified according to ANCA positivity (ANCA + vs. ANCA−). **e** IL-1β levels. **f** IL-18 levels. **g** CCL-23 levels. **h** VEGF levels. Data are expressed as the median and interquartile range (IQR). p was calculated according to Mann-Whitney test: *p < 0.05, **p < 0.01. Abbreviations are as follows: GPA, granulomatosis with polyangiitis; EGPA, eosinophilic granulomatosis with polyangiitis; MPA, microscopic polyangiitis

To explore inflammatory and cell death–related pathways potentially involved in vascular injury in AAV, we measured a selected panel of cytokines mainly associated with inflammasome activation and cell death, together with CCL23 and VEGF, a chemokine and a growth factor relevant to vascular inflammation and endothelial activation. Plasma levels of cytokines, chemokines and VEGF are summarized in Fig. 2. IL-1β was significantly elevated in EGPA (*p* < 0.05) and MPA (*p* < 0.01) versus HDs. IL-18 levels were higher in all patient groups (GPA and EGPA, *p* < 0.05, *p* < 0.01; MPA, *p* < 0.0001) than in HDs, and MPA had higher IL-18 levels than EGPA (*p* < 0.05). CCL-23 was elevated in GPA and MPA compared to EGPA and HDs, while VEGF was significantly higher only in MPA versus HDs (*p* < 0.01). TNFα and IL-1α were undetectable across groups. The lack of measurable TNFα and IL-1α likely reflects biological and technical constraints rather than the complete absence of inflammatory activity.

In EGPA, ANCA-positive patients exhibited higher IL-1β (*p* < 0.05) and CCL-23 plasma levels (*p* < 0.01) compared to ANCA-negative patients. Additionally, in ANCA-positive EGPA patients, neutrophil percentages positively correlated with IL-1β (*r* = 0.818, *p = 0.019) and CCL-23 levels (*r* = 0.664, *p = 0.036) (Online Resource 3).

### PBMC cell death induced by EGPA plasma

**Fig. 3:**
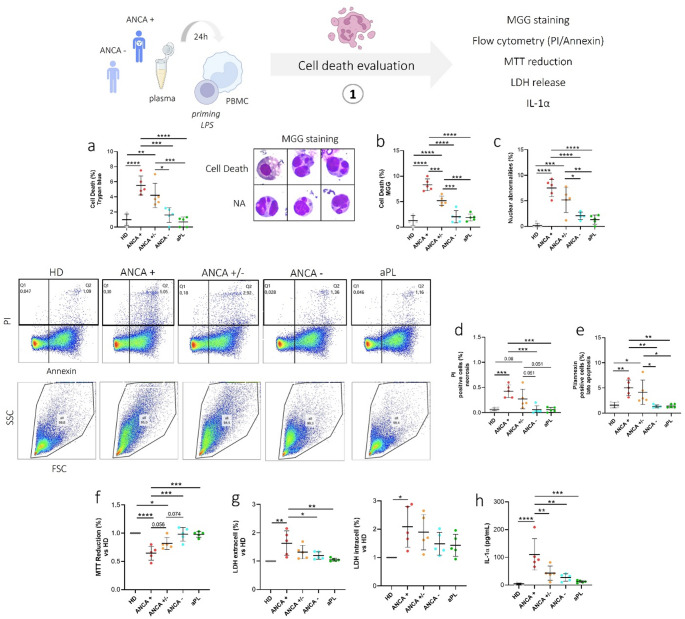
Cell death and nuclear abnormalities in PBMCs treated with plasma from EGPA patients. PBMCs were treated as described in Materials and Methods with 10% of plasma from EGPA patients (ANCA+, ANCA+/−, ANCA-), patients positive to apL and HDs. **a** Rate of cell death, Trypan blue assay. **b** Rate of cell death, MGG staining. **c** Rate of NA, MGG staining. **d** Percentage of PBMCs positive for PI (Q1). **e** Percentage of PBMCs positive for PI/Annexin (Q2). **f** MTT reduction. **g** Extracellular and intracellular LDH levels. **h** IL-1α levels. Data are shown as the media (SD). p calculated according to the One Way Anova test: *p < 0.05, **p < 0.01, ***p < 0.01, ****p < 0.001. Abbreviations are as follows: HD, healthy donors; ANCA, Anti-neutrophil cytoplasmic antibodies; aPL, antiphospholipid antibodies; NA, nuclear abnormalities

We evaluated the effect of plasma from ANCA+, ANCA+/− and ANCA− EGPA patients on PBMCs from HDs. Plasma from ANCA + patients induced significantly increased PBMC death after 24 h compared to ANCA− patients (*p* < 0.001), aPL controls (*p* < 0.0001), and HDs (*p* < 0.0001). Increased cell death was also observed in PBMCs stimulated with ANCA+/− plasma compared to those stimulated with ANCA− plasma (*p* = 0.011) aPL plasma (*p* < 0.001) and HDs plasma (*p* < 0.01) (Fig. 3a). These findings were further confirmed by MGG cytological analysis (Fig. 2b). Pyknosis and karyorrhexis were higher in PBMCs treated with ANCA + plasma than in PBMCs treated with ANCA+/− (*p* < 0.001), ANCA− (*p* < 0.0001), aPL (*p* < 0.0001) and HDs (*p* < 0.0001) plasma. Increased pyknosis and karyorrhexis were also observed in PBMCs stimulated with ANCA+/− plasma compared to those stimulated with ANCA− (*p* < 0.001), aPL (*p* < 0.001) and HDs plasma (*p* < 0.0001) (Fig. 3b).

Additionally, nuclear abnormalities were higher in PBMCs treated with ANCA + plasma than in those treated with ANCA− (*p* < 0.0001), aPL (*p* < 0.0001), or HDs (*p* < 0.0001) plasma (Fig. 3c). Increased nuclear abnormalities were also observed in PBMCs stimulated with ANCA+/− plasma compared to those stimulated with ANCA− (*p* < 0.05), apL (*p* < 0.01) and HDs plasma (*p* < 0.001) (Fig. 3c).

Flow cytometry showed higher percentage of necrotic (PI-positive) and late apoptotic (Annexin/PI-positive) PBMCs upon exposure to ANCA + and ANCA+/− plasma compared to ANCA−, aPL, or HD plasmas (Fig. 3d, e).

PBMC metabolic activity (MTT reduction) was lower following stimulation with ANCA + plasma compared to all other groups (Fig. 3f, g).

Extracellular LDH levels, that reflect membrane damage and necrosis, were higher in PBMCs treated with plasma from ANCA + patients than those treated with plasma from ANCA− (*p* < 0.05), aPL patients (*p* < 0.01) and HDs (*p* < 0.01). No significant differences in intracellular LDH levels were detected across all groups (Fig. 3g).

Moreover, IL-1α secretion was notably increased in PBMCs supernatant stimulated with plasma from ANCA + patients (Fig. 3h). Correlation analysis between the different cell death measurements, and the resulting correlation matrix is provided in the Supplementary Materials (Online Resource 4). This analysis shows a strong concordance between flow cytometry, MGG staining, Trypan blue exclusion, and MTT assays, confirming the overall consistency of the results obtained with these complementary methods.

### Inflammasome: dependent cytokine release in PBMCs induced by EGPA plasma

**Fig. 4:**
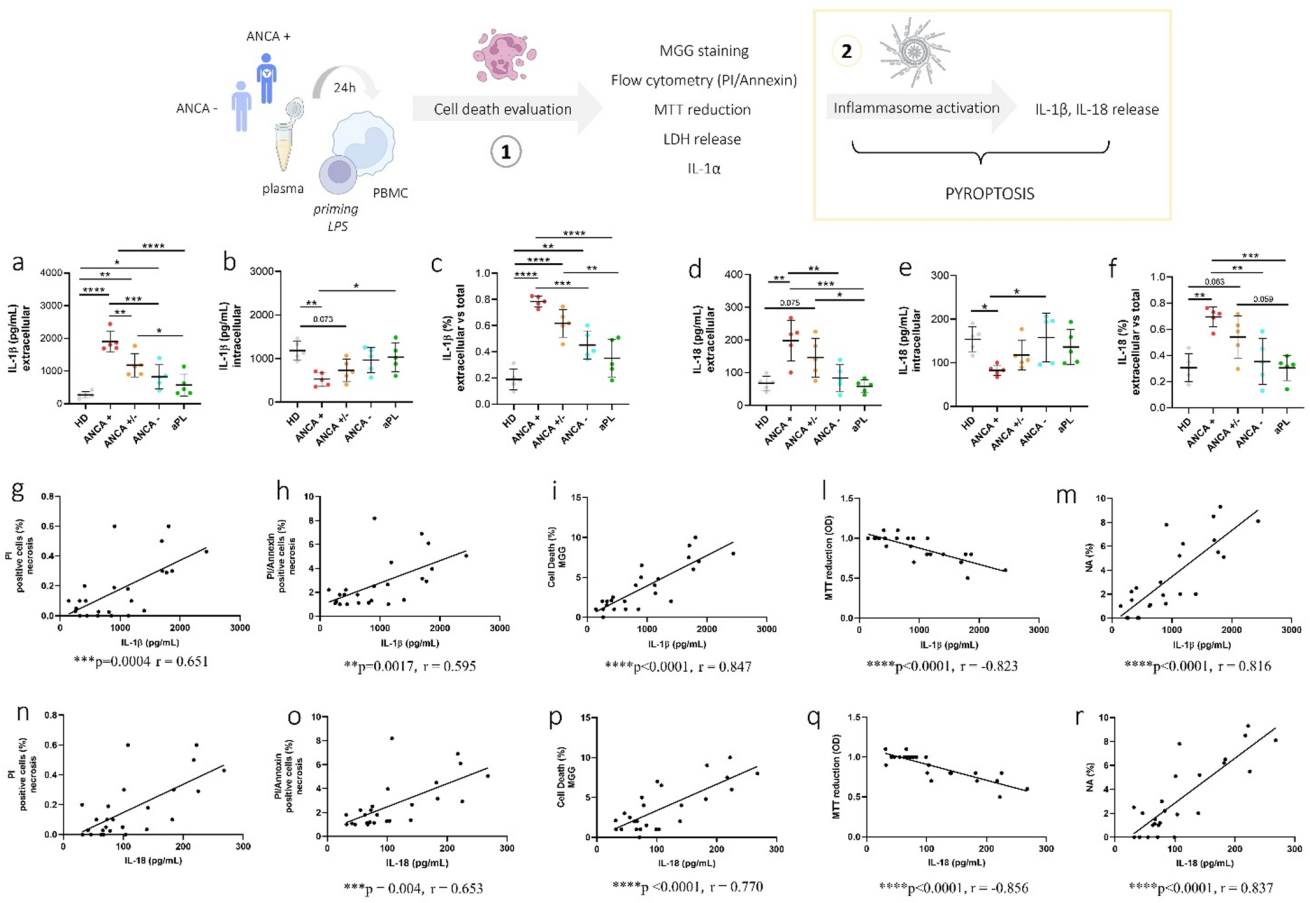
IL-1β and IL-18 release in PBMCs treated with plasma from EGPA patients. IL-1β and IL-18 formation and release in PBMCs treated with plasma from ANCA + and ANCA− EGPA. PBMCs were treated as described in Materials and Methods with 10% of plasma from EGPA patients (ANCA+, ANCA+/−, ANCA-), patients positive to aPL and HDs. **a** Extracellular IL-1β levels. **b** Intracellular IL-1β levels. **c** IL-1β release vs. total IL-1β production. **d** Extracellular IL-18 levels. **e** Intracellular IL-18 levels. **f** IL-18 release vs. total IL-1β production. Data are shown as the media (SD). p calculated according to the One Way Anova test: *p < 0.05, **p < 0.01, ***p < 0.001, ****p < 0.0001. Inflammasome-related cytokines correlate with cell death and MTT activity. **g**–**m** IL-1β levels correlate with the percentage of PBMCs positive for PI and PI/Annexin V, the rate of cell death (MGG staining), MTT reduction, and the rate of NA observed by MGG staining. **n**–**r** IL-18 levels correlate with the percentage of PBMCs positive for PI and PI/Annexin V, the rate of cell death (MGG staining), MTT reduction, and the rate of NA observed by MGG staining. Pearson correlation test. Abbreviations are as follows: HD, healthy donors; ANCA, Anti-neutrophil cytoplasmic antibodies; aPL, antiphospholipid antibodies; MGG, May-Grünwald Giemsa; PI, propidium iodide; NA, nuclear abnormalities

PBMCs treated with plasma from ANCA + patients released significantly more IL-1β than those exposed to plasma from ANCA−, aPL patients, and HDs. Additionally, PBMCs treated with plasma from ANCA+/− patients released more IL-1β than those exposed to plasma from aPL patients and HDs (Fig. 4a–c). IL-18 secretion showed a similar trend, without reaching statistical significance, when comparing ANCA + versus ANCA−, healthy donors (HD), and aPL patients (Fig. 4d–f). IL-1β and IL-18 release positively correlated with cell death markers, nuclear abnormalities, and inversely correlated with MTT reduction (Fig. 4g–m).

### Chemotactic factor production and PBMC migration following stimulation with EGPA plasma

**Fig. 5:**
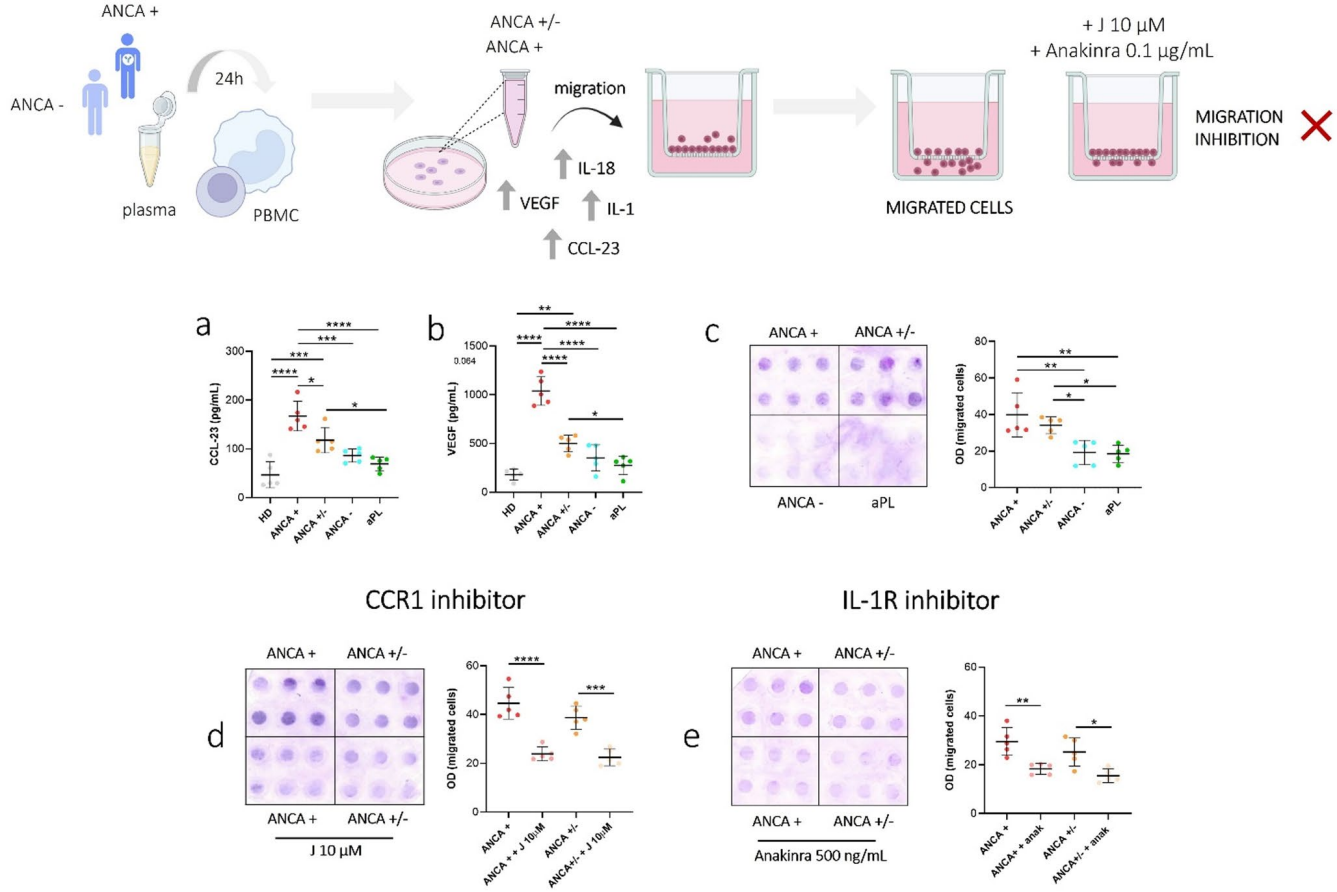
Effect of plasma from EGPA patients on chemokines, growth factors levels and cell migration. VEGF and CCL-23 formation and release in PBMCs treated with plasma from ANCA + and ANCA− EGPA. PBMCs were treated as described in Materials and Methods with 10% of plasma from EGPA patients (ANCA+, ANCA+/−, ANCA−), patients positive to apL and HDs. **a** CCL-23 levels. **b** VEGF levels. **c** Effect of conditioned media on PBMCs migration. Cell migration is shown as optical density values (O.D). Each sample was tested in sextuplicate. **d** Effect of 10 µM J113863 on PBMCs migration induced by conditioned media. **e** Effect of anakinra on PBMCs migration induced by conditioned media. p calculated according to the One Way Anova test: *p < 0.05, **p < 0.01, ***p < 0.001, ****p < 0.0001. Abbreviations are as follows: HD, healthy donors; ANCA, Anti-neutrophil cytoplasmic antibodies; aPL, antiphospholipid antibodies

Chemotactic factors such as VEGF and CCL-23 were measured to assess their release from PBMCs following stimulation with plasma from EGPA patients. PBMC stimulation with ANCA + EGPA plasma significantly increased VEGF and CCL-23 release compared to ANCA−, aPL patients, and HDs (Fig. 5a-b). Additionally, CCL-23 and VEGF levels were elevated in PBMCs stimulated with plasma from ANCA+/− patients compared to those stimulated with plasma from apL patients and HDs (Fig. 5b).

Conditioned media from PBMCs stimulated with ANCA + and ANCA+/− plasma induced significantly greater PBMCs migration compared to other groups (Fig. 5c). PBMC migration was markedly inhibited by interleukin-1β receptor antagonist (anakinra) and with the CCR1 chemokine receptor inhibitor (J113863) supporting the hypothesis that the conditioned media induce PBMCs migration through the release of IL-1β and chemokines (Fig. 5d-e). Migration positively correlated with IL-1α (*p* < 0.0001, *r* = 0.846), VEGF (*p* < 0.0001, *r* = 0.766), CCL-23 (*p* = 0.001, *r* = 0.753) IL-1β (*p* = 0.013, *r* = 0.546) and IL-18 (*p* = 0.001, *r* = 0.674) levels (Online Resource 5).

## Discussion

In AAV patients in clinical remission, peripheral blood cytology revealed persistent disease-associated abnormalities in neutrophil morphology and maturation. Our findings of elevated circulating immature neutrophils support the hypothesis of sustained innate immune activation even after clinical remission has been achieved. These results align with previous studies reporting the presence of circulating immature neutrophils across all AAV subtypes, likely associated to sustained myelopoietic stimulation driven by ongoing subclinical inflammation [33, 34]. Interestingly, only a minority of patients showed a moderate left shift on the I/T ratio, indicating a predominantly qualitative rather than quantitative inflammatory response. We found that GPA patients exhibited increased neutrophil counts, along with more hyposegmented neutrophils and nuclear abnormalities, suggesting enhanced neutrophil activation and turnover. This pattern is consistent with the relapsing-remitting course typical of GPA [35]. In contrast, EGPA and MPA patients showed a significant increase in hypersegmented neutrophils compared to HDs, often linked to prolonged circulation time or a recovery from inflammation, potentially reflecting distinct disease trajectories [32]. Our findings are consistent with previous studies reporting biochemical and functional evidence of ongoing neutrophil priming and neutrophil extracellular trap (NET) formation during remission, suggesting that immunological quiescence is more accurately described as “quiet” rather than truly “off” [16, 17]. Persistent monocyte activation has also been reported in AAV, as evidenced by sustained expression of activation markers such as CD11b after clinical remission [19]. Although we did not observe major abnormalities in monocytes or lymphocytes in our cohort, apart from an increased vacuolization in monocytes from ANCA-positive EGPA patients.

In addition, our data show that plasma levels of IL-1β and IL-18 were modestly elevated in all AAV groups compared with healthy controls. Although not dramatically high, these increases further indicate the persistence of subtle inflammatory signals during clinical remission, likely linked to inflammasome activation (Fig. 2). A recent follow-up study demonstrated that IL-18BP is significantly associated with disease activity in AAV. Our finding of increased IL-18 levels even in patients in clinical remission is consistent with this observation, as elevated IL-18 concentrations are often accompanied by increased levels of its natural inhibitor, IL-18BP. This suggests that IL-18 may represent a useful marker for assessing relapse risk, potentially offering an advantage over IL-1β, which is less readily detectable in the plasma or serum of patients [36]. Moreover, higher levels of CCL23 and VEGF were detected across the different patient groups compared with healthy controls (Fig. 2). Recent studies have reported that increased concentrations of CCL23 and VEGFA correlate with active disease in AAV and may serve as biomarkers for predicting disease relapse [37, 38] Therefore, the presence of these two biomarkers could be further evaluated and validated as potential indicators of relapse risk in patients with AAV who appear to be in clinical remission.

Cell death is likely involved in the release of damage-associated molecular patterns and enhance the presence of the autoantigen at the site of inflammation [14]. Our cytological analyses revealed higher cellular mortality in AAV samples compared to HDs. A particularly interesting finding pertains to EGPA patients. Specifically, ANCA-positive patients exhibited higher leukocyte mortality levels (Fig. 1) and higher IL-1β and CCL-23 plasma levels (Fig. 2) compared to ANCA-negative EGPA patients. Interestingly, the percentage of neutrophils positively correlated with plasma levels of IL-1β and CCL-23 in ANCA-positive EGPA patients, but not in ANCA-negative patients, suggesting that ANCA positivity may be associated with a higher pro-inflammatory profile.

These observations prompted us to further investigate the immunological effects of plasma from EGPA patients in remission on PBMCs in vitro, with a particular focus on both ANCA-positive and ANCA-negative individuals. Our choice to study EGPA was not driven by stronger cytological abnormalities compared with GPA or MPA, but rather by the unique biological heterogeneity of this subtype, which provides a naturally occurring framework to explore how ANCA status may influence subclinical immune activation during remission, an aspect not addressable in GPA or MPA, where most patients are ANCA-positive.

Our study revealed that EGPA patients display distinct immune profiles depending on their ANCA status. Plasma from ANCA-positive EGPA patients induced higher cell death, nuclear abnormalities and inflammasome-derived cytokine (IL-1β, IL-18) release from PBMCs compared to ANCA-negative EGPA plasma (Figs. 3 and 4). It is possible that the release of IL-1β, together with the release of DAMPs associated with immunogenic cell death, could, in vivo, contribute to neutrophil priming and thereby amplify inflammatory responses in AAV. Importantly, IL-1β can also induce the production of other pro-inflammatory cytokines and promote neutrophil degranulation, further reinforcing its role as a key mediator in AAV pathogenesis and connecting inflammasome activation to the persistence of subclinical inflammation during remission [11, 39]. Finally, the percentage of cell death, reduced metabolic activity and nuclear abnormalities strongly correlated with levels of inflammasome-related cytokines, suggesting that the observed cell death in our experiments may, at least in part, result from inflammasome activation consistent with pyroptosis.

A direct comparison of these two datasets is not straightforward, as the cytological data were generated from patients’ own leukocytes, whereas the in vitro experiments were performed using PBMCs from healthy donors exposed to patient plasma. Nevertheless, we also show a correlation between the degree of leukocyte cell death in patients and the extent of cell death observed in the PBMC assays, further supporting the link between the two observations (Online Resource 6). Our findings add an additional layer of complexity to previous research, which reported differences between ANCA-positive and ANCA-negative EGPA patients in other experimental contexts. For example, Natorska et al. found that plasma from EGPA patients in remission significantly increased NET formation in healthy neutrophils independently of ANCA status, but correlated positively with peripheral eosinophil counts [40]. Our contrasting observation, highlighting clear differences linked to ANCA positivity, suggests a nuanced interplay between eosinophil-driven and ANCA-driven inflammatory mechanisms in EGPA, further underlining the disease’s biological heterogeneity.

Indeed, the clinical differentiation of ANCA-positive and ANCA-negative EGPA aligns with genetic studies demonstrating divergent genetic architectures [41]. ANCA-positive EGPA patients exhibit genetic and clinical profiles closely resembling those of classical AAV (GPA / MPA), often presenting with more prominent vasculitic features such as glomerulonephritis and peripheral neuropathy [4, 42]. Moreover, they display a higher propensity for relapse compared to ANCA-negative EGPA patients, though this excess risk is less pronounced compared to the differences seen between PR3-ANCA and MPO-ANCA positive patients within classic GPA and MPA cohorts [43]. In contrast, ANCA-negative EGPA tends to manifest primarily through eosinophil-driven inflammation, predominantly affecting respiratory and ENT compartments [4, 42].

Finally, plasma levels of CCL-23 and VEGF, key mediators of eosinophil/monocyte recruitment and vascular remodeling, were significantly elevated, supporting their role in EGPA-related tissue eosinophilia and vascular inflammation (Fig. 2). Conditioned media from PBMCs exposed to plasma from ANCA-positive EGPA patients enhanced PBMC migration (Fig. 5), which correlated with IL-1β, IL-1α, IL-18, CCL-23, and VEGF levels, suggesting that these mediators collectively contribute to immune cell recruitment and sustained vascular inflammation [44, 45].

While our study provides novel insights into the potential role of IL-1β, IL-18, VEGF, and CCL-23 as biomarkers in AAV, several limitations should be considered. The relatively small cohort may limit the generalizability of our findings, and causal relationships cannot be inferred from the observed associations.

Functional analyses were restricted to PBMCs, and ongoing work extending these studies to neutrophils will clarify whether similar mechanisms influence neutrophil activity. Moreover, the study focused exclusively on patients in remission, without a direct comparison to those with active disease, and the sample size was insufficient for detailed subgroup analyses based on AAV phenotypes, treatment regimens, or duration of remission.

These limitations directly inform the future directions of our research. Longitudinal studies are warranted to evaluate the predictive value of IL-1β, IL-18, VEGF, and CCL-23 as biomarkers of relapse in patients with AAV, and to validate this cytokine panel in larger, independent cohorts. Moreover, the mechanistic insights obtained from PBMC analyses will be extended to neutrophils to determine whether the pathways identified also modulate neutrophil activity. These cytological studies will be complemented by the assessment of neutrophil activation and maturation markers, providing a more comprehensive understanding of neutrophil functional status and its potential contribution to disease pathophysiology. Importantly, these analyses will also be conducted in patients with GPA and MPA in clinical remission to assess whether the same mechanisms are active in these populations. Collectively, these approaches aim to translate the current findings into clinically relevant tools for risk stratification and targeted therapeutic interventions in AAV.

## Conclusion

Our study provides novel evidence supporting the concept of persistent subclinical immune activation during remission in AAV, with a particular focus on EGPA. Specifically, in ANCA-positive EGPA, our findings suggest that residual immune activity may be associated with PBMC activation and inflammasome-related signaling even in the absence of overt clinical activity. While eosinophils are classically implicated in EGPA pathogenesis, the observed PBMC activation and proinflammatory cytokine release indicate that additional immune pathways may also be engaged, particularly in ANCA-positive patients. From a clinical standpoint, the capacity of plasma from EGPA patients in clinical remission to induce PBMC activation highlights the limitations of conventional clinical and serologic markers in fully capturing true disease remission. This is particularly relevant in EGPA, where symptoms may be minimal despite ongoing immunologic activation. Biomarker-based monitoring using IL-1β, IL-18, VEGF, and CCL-23 may thus complement current clinical and serologic criteria and provide earlier warning signs of impending relapse.

Additionally, the distinct immune profiles observed between ANCA-positive and ANCA-negative EGPA are consistent with the notion that these subgroups represent overlapping yet biologically distinct entities, which may inform personalized therapeutic strategies in the future. ANCA status could therefore help guide personalized therapeutic strategies, particularly in patients with persistent immunologic activity despite apparent remission. Finally, the role of mononuclear cells, inflammasome activation, and immunogenic cell death in ANCA-positive EGPA represents a promising area for further investigation and therapeutic innovation.

## Supplementary Information

Below is the link to the electronic supplementary material.

Supplementary Material 1

## Data Availability

Data will be made available on request.
