# Supplementary material for: Immature leukocyte and plasma-induced cell death reveal subclinical immune activation in EGPA patients in remission
Source: Inflamm Res. 2026 Feb 2;75(1):31. doi: 10.1007/s00011-026-02189-7 (PMC12862012; doi:10.1007/s00011-026-02189-7)
Supplement: Supplementary file 1 — Supplementary Material 1 [file 11_2026_2189_MOESM1_ESM.docx]

**Immature Leukocyte and Plasma-Induced Cell Death Reveal Subclinical Immune Activation in EGPA Patients in Remission**

Chiara Baggio^a^*, Luca Iorio^a^, Carlotta Boscaro^b,c^, Federica Davanzo^a^, Veronica Davanzo^d^, Michela Pelloso^d^, Marta Tonello^a^, Paolo Sfriso^a^, Mattia Albiero^e^, Roberta Ramonda^a^, Andrea Doria^a^, Roberto Padoan^a†^, Francesca Oliviero^a†^*

^a^ Rheumatology Unit, Department of Medicine - DIMED, University of Padova, Padova, Italy

^b^ Department of Medicine - DIMED, University of Padova, Padova, Italy

^c^ Veneto Institute of Molecular Medicine, Laboratory of Experimental Diabetology, Padova, Italy

^d^ Department of Laboratory Medicine, University-Hospital of Padova, Padova, Italy

^e^ Department of Surgery, Oncology, and Gastroenterology, University of Padova, Padova, Italy

**Corresponding authors:** Chiara Baggio, Francesca Oliviero

Rheumatology Unit, Department of Medicine - DIMED, University of Padova, Padova, Italy.

e-mail: [chiara.baggio@unipd.it](mailto:chiara.baggio@unipd.it), [francesca.oliviero@unipd.it](mailto:francesca.oliviero@unipd.it)

Phone: +39 049 8218682

**Online Resource 1.** Leukocyte morphological features, definitions, and functional interpretations.

| **Feature** | **Morphology** | **Biological Significance** |
| --- | --- | --- |
| **Vacuoles** (Figure 1e, g)  *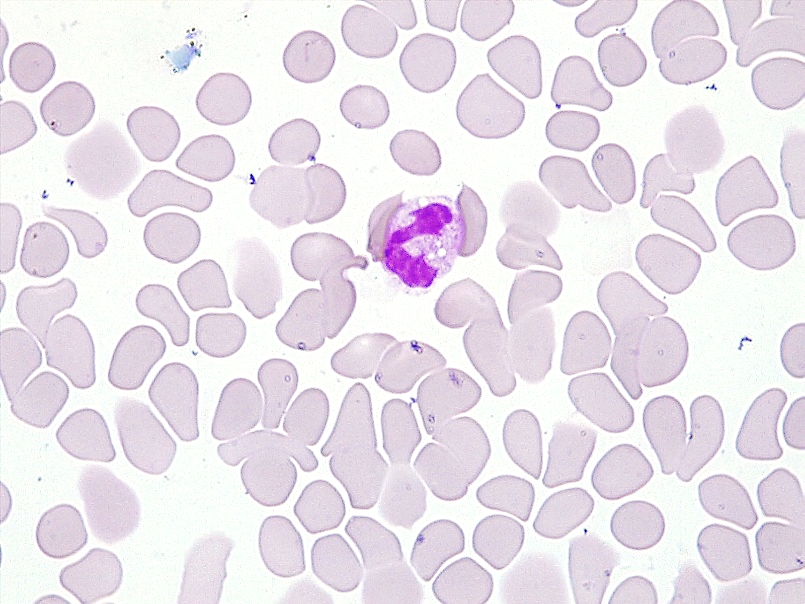 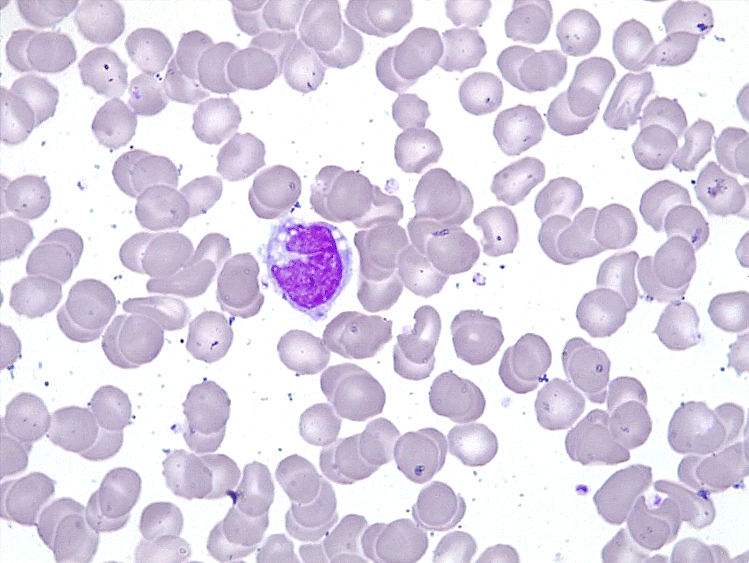* | Cytoplasmic clear spaces within leukocytes, often appearing as rounded, unstained areas in the cytoplasm | Cellular stress or degeneration |
| **Micronuclei** (Figure 1l)  **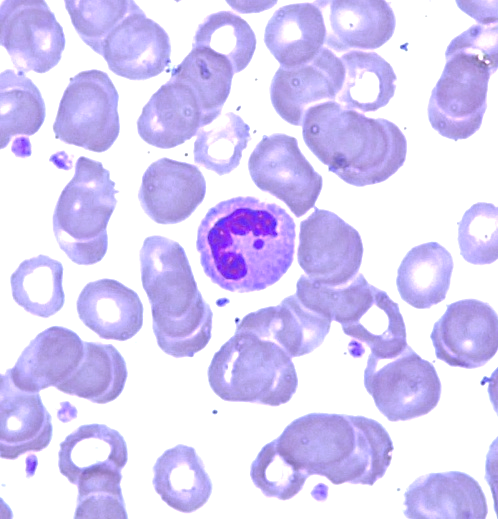** | A round cytoplasmic inclusion having a diameter one-tenth to one-third that of the primary nucleus, containing intact or fragments of chromosomes | DNA damage and chromosomal instability |
| **Buds** (Figure 1l)  **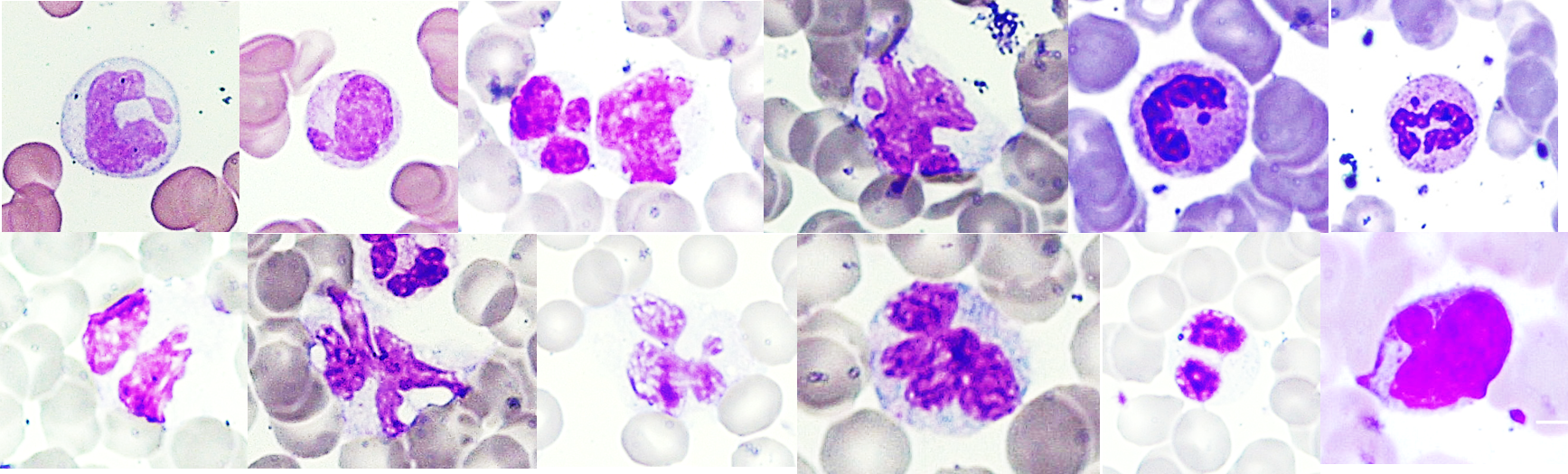** | Small nuclear protrusions | DNA damage and chromosomal instability |
| **Binucleated cells** (Figure 1m)  *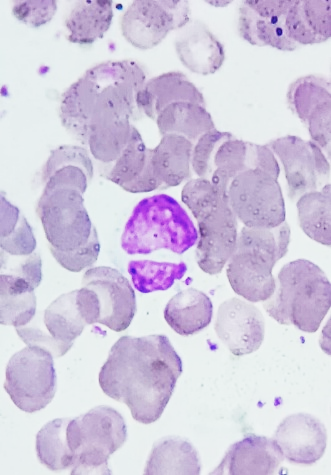* | Cells with two nuclei | Cytokinesis defects or abnormal cell division |
| **Karyorrhectic cells** (Figure 1n)  **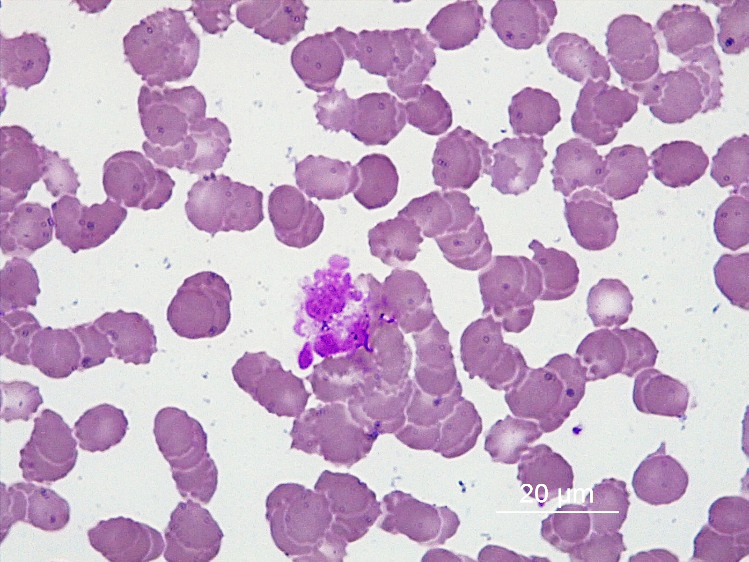** | Disintegration of nuclear chromatin into separate structures | Advanced apoptosis or necrosis |
| **Karyolytic cells** (Figure 1n)  **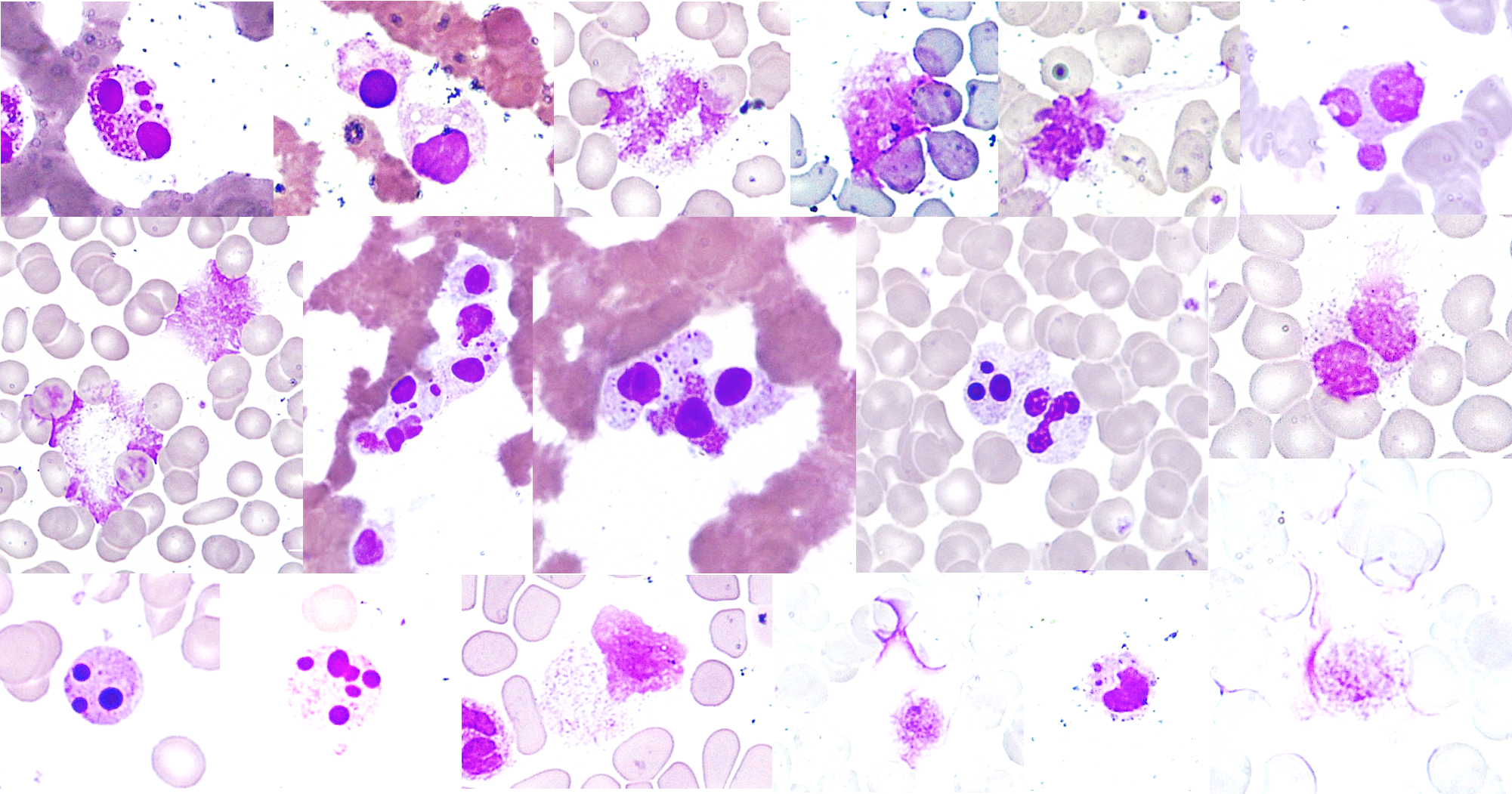** | Loss of chromatin staining ability and its dissolution. Uniformly eosin-stained, ghost-like nucleus | Very late-stage cell death / necrosis |
| **Pyknosis** (Figure 1n)  **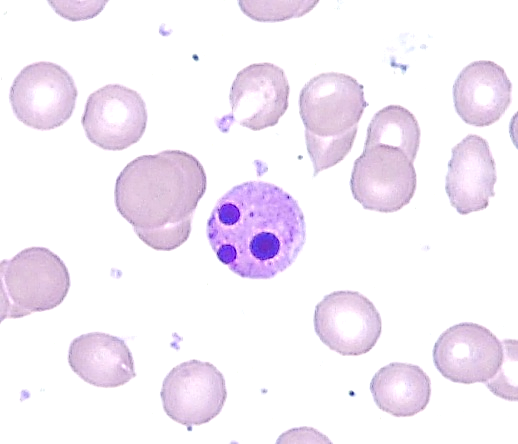** | Irreversible condensation of chromatin. Nuclear shrinkage, condensed chromatin | Apoptosis or early necrosis |
| **Left shift** (Figure 1a)  **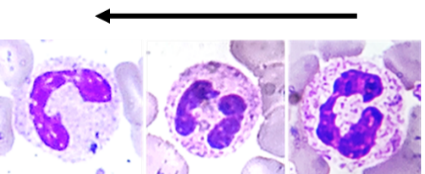** | The presence of immature neutrophils | Reflecting bone marrow response to stress or inflammation |
| **Right shift** (Figure 1d)  **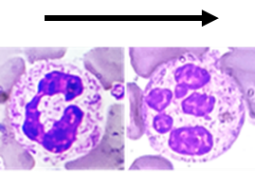** | The presence of hypersegmented neutrophils | Normal response to recovery from an infection or inflammation |

**Online Resource 2. Cytological analysis of EGPA patients stratified by ANCA status at diagnosis.**

|  | **ANCA positive** | **ANCA negative** | **p** |
| --- | --- | --- | --- |
| **N, median (IQR)** | 63.2 (54-64) | 69.8 (54-73) | 0.984 |
| **Hyposegmented N, median (IQR)** | 8.5 (5.6-13) | 9.5 (5.5-13.5) | 0.960 |
| **Band N, median (IQR)** | 4.0 (2.5-6.6) | 4.8 (3.0-8.6) | 0.301 |
| **Hypersegmented N, median (IQR)** | 2.2 (0.8-3.2) | 1.5 (0.6-3.4) | 0.638 |
| **N with vacuoles, median (IQR)** | 0 (0.0-1.3) | 0.0 (0.0-0.6) | 0.511 |
| **M, median (IQR)** | 7.4 (5.4-9.7) | 6.1 (4.6-9.4) | 0.429 |
| **M with vacuoles, median (IQR)** | 1.3 (0.7-3.4)* | 0.6 (0.0-1.2) | 0.029 |
| **L, median (IQR)** | 22.3 (17-29) | 20.3 (18-32) | 0.913 |
| **Eosinophils + Basophils, median (IQR)** | 5.8 (0.0-9.5) | 1.7 (0.0-10) | 0.927 |
| **N with NA, median (IQR)** | 0.0 | 0.0 | 0.887 |
| **Binucleated M, median (IQR)** | 0.5 (0.0-1.4) | 0.0 (0.0-0.8) | 0.522 |
| **Cell Death, median (IQR)** | 1.6 (0.6-2.7)** | 0.0 (0.0-0.8) | 0.007 |

Data are expressed as the median and interquartile range (IQR). p was calculated according to Mann-Whitney test. Abbreviations are as follows: EGPA, eosinophilic granulomatosis with polyangiitis; ANCA, Anti-neutrophil cytoplasmic antibodies; N, neutrophils; M, monocytes; L, lymphocytes; NA, nuclear abnormalities; IQR, interquartile range.

**Online Resource 3. Correlation between cytokine, chemokines, growth factors and cytological analysis.**

|  |  | **N, %** | **M, %** | **L, %** |
| --- | --- | --- | --- | --- |
| **EGPA ANCA+** | **IL-1β, pg/mL** | *p = 0.019, r = 0.818 | *p = 0.013, r = -0.706 | **p = 0.004, r = -0.774 |
|  | **IL-18, pg/mL** | p = 0.619, r = 0.161 | p = 0.266 , r = -0.350 | p = 0.09, r = -0.515 |
|  | **CCL-23, pg/mL** | *p = 0.036, r = 0.664 | *p = 0.017, r = -0.699 | p = 0.082, r = -0.525 |
| **EGPA ANCA**− | **IL-1β, pg/mL** | p = 0.091, r = 0.390 | p = 0.867, r = 0.041 | p = 0.405, r = -0.203 |
|  | **IL-18, pg/mL** | p = 0.505, r = 0.163 | p = 0.811, r = 0.059 | p = 0.875 , r = 0.039 |
|  | **CCL-23, pg/mL** | p = 0.060, r = 0.437 | p = 0.569, r = -0.140 | p = 0.175, r = -0.325 |

Spearman’s rank correlation test. *p<0.05, **p<0.01. Abbreviations are as follows: GPA, granulomatosis with polyangiitis; EGPA, eosinophilic granulomatosis with polyangiitis; N, neutrophils; M, monocytes; L, lymphocytes.

**Online Resource 4. Correlation between cell death parameters.**

|  | **Cell death**  **MGG, %** | **Cell death**  **Trypan blue, %** | **MTT reduction, OD** | **LDH release, OD** | **IL-1α,**  **pg/mL** |
| --- | --- | --- | --- | --- | --- |
| **PI, %** | ****p<0.0001  r = 0.853 | ****p<0.0001  r = 0.901 | ****p<0.0001  r = -0.798 | **p = 0.0055  r = 0.5383 | ****p<0.0001  r = 0.726 |
| **PI/Annexin, %** | ****p<0.0001  r = 0.786 | ****p<0.0001  r = 0.868 | ****p<0.0001  r = -0.786 | **p = 0.0044  r = 0.5503 | ***p=0.0006  r = 0.640 |
| **Cell death (MGG), %** | **/** | ****p<0.0001  r = 0.938 | ****p<0.0001  r = -0.841 | p = 0.0625  r = 0.3779 | ****p<0.0001  r = 0.835 |
| **Cell death (Trypan blue), %** | ****p<0.0001  r = 0.938 | **/** | ****p<0.0001  r = -0.847 | *p = 0.0179  r = 0.4694 | ****p<0.0001  r = 0.7669 |
| **MTT reduction (OD)** | ****p<0.0001  r = -0.841 | ****p<0.0001  r = -0.847 | **/** | p = 0.1388  r = -0.3046 | ****p<0.0001  r = -0.796 |

Pearson correlation test. Abbreviations are as follows: MGG, May-Grünwald Giemsa; PI, propidium iodide; OD, optical density.

**Online Resource 5. Cytokines, CCL-23 and VEGEF levels correlates with cell migration.**

|  | **Cell migration (OD)** |
| --- | --- |
| **IL-1β, pg/mL** | *p = 0.013, r = 0.546 |
| **IL-1α, pg/mL** | ****p<0.0001, r = 0.846 |
| **IL-18, pg/mL** | **p = 0.001, r = 0.674 |
| **CCL-23, pg/mL** | ***p = 0.001, r = 0.753 |
| **VEGF, pg/mL** | ****p<0.0001, r = 0.766 |

Pearson correlation test. Abbreviations are as follows: OD, optical density.

**Online Resource 6. Correlation between in vitro and ex vivo cell death outcomes**


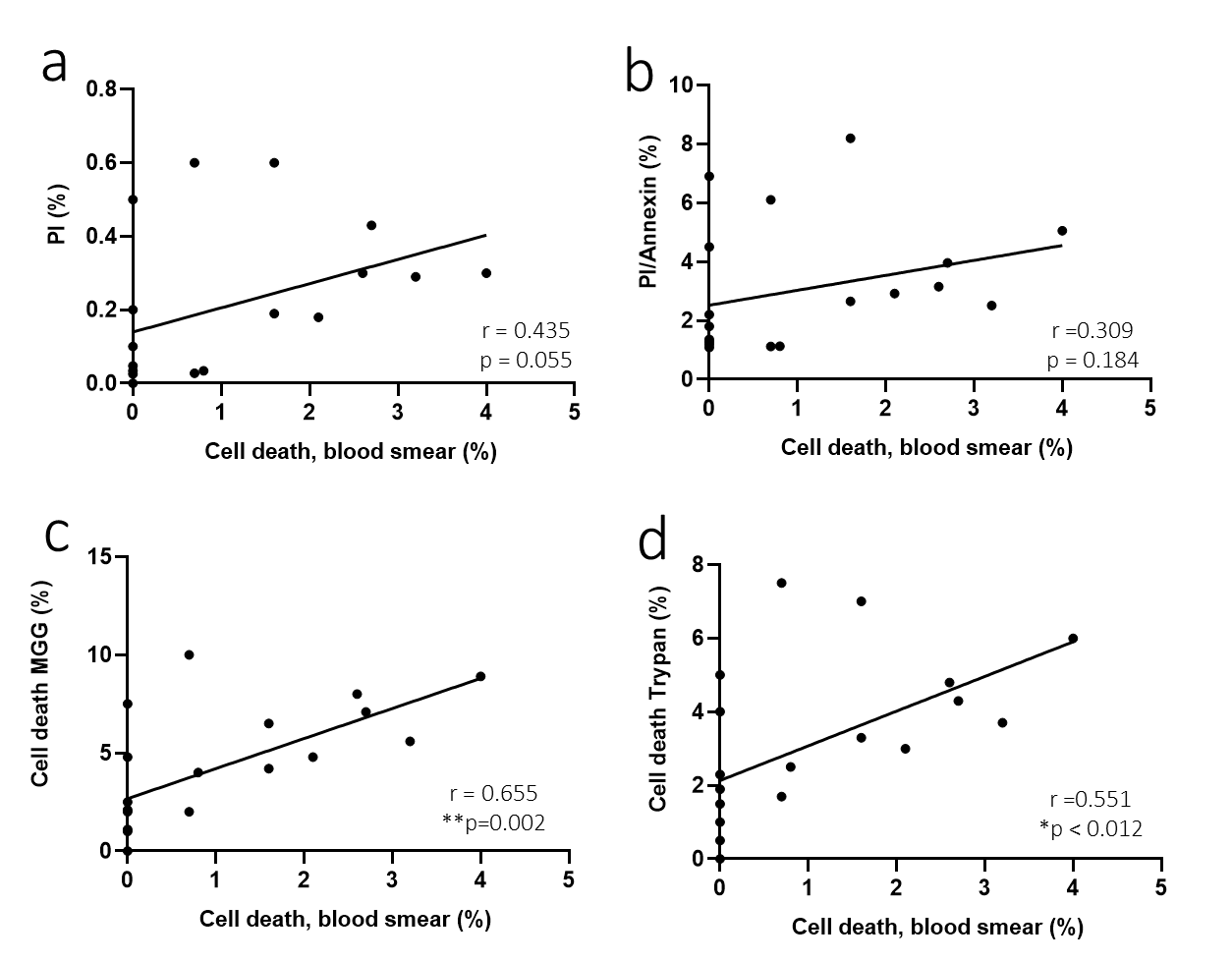


Pearson correlation test. Abbreviations are as follows: MGG, May-Grünwald Giemsa; PI, Propidium.
